# Concomitant contraceptive implant and efavirenz use in women living with HIV: perspectives on current evidence and policy implications for family planning and HIV treatment guidelines

**DOI:** 10.7448/IAS.20.1.21396

**Published:** 2017-05-11

**Authors:** Rena C. Patel, Chelsea Morroni, Kimberly K. Scarsi, Tabitha Sripipatana, James Kiarie, Craig R. Cohen

**Affiliations:** ^a^ Division of Allergy and Infectious Diseases, Department of Medicine, University of Washington, Seattle, WA, USA; ^b^ Wits Reproductive Health and HIV Institute, University of the Witwatersrand, Johannesburg, South Africa; ^c^ Institutes for Women’s Health and Global Health, University College London, London, UK; ^d^ Botswana-UPenn Partnership, University of Botswana, Gaborone, Botswana; ^e^ College of Pharmacy, University of Nebraska Medical Center, Omaha, NE, USA; ^f^ Bureau for Global Health, Office of Population & Reproductive Health, USAID, Washington, DC, USA; ^g^ Department of Reproductive Health and Research, World Health Organization, Geneva, Switzerland; ^h^ Bixby Center for Global Reproductive Health, University of California San Francisco, San Francisco, CA, USA

**Keywords:** contraceptive implants, efavirenz, pregnancy, HIV-positive women, policy

## Abstract

**Introduction**: Preventing unintended pregnancies is important among all women, including those living with HIV. Increasing numbers of women, including HIV-positive women, choose progestin-containing subdermal implants, which are one of the most effective forms of contraception. However, drug–drug interactions between contraceptive hormones and efavirenz-based antiretroviral therapy (ART) may reduce implant effectiveness. We present four inter-related perspectives on this issue.

**Discussion**: First, as a case study, we discuss how limited data prompted country-level guidance against the use of implants among women concomitantly using efavirenz in South Africa and its subsequent negative effects on the use of implants in general. Second, we discuss the existing clinical data on this topic, including the observational study from Kenya showing women using implants plus efavirenz-based ART had three-fold higher rates of pregnancy than women using implants plus nevirapine-based ART. However, the higher rates of pregnancy in the implant plus efavirenz group were still lower than the pregnancy rates among women using common alternative contraceptive methods, such as injectables. Third, we discuss the four pharmacokinetic studies that show 50–70% reductions in plasma progestin concentrations in women concurrently using efavirenz-based ART as compared to women not on any ART. These pharmacokinetic studies provide the biologic basis for the clinical findings. Fourth, we discuss how data on this topic have marked implications for both family planning and HIV programmes and policies globally.

**Conclusion**: This controversy underlines the importance of integrating family planning services into routine HIV care, counselling women appropriately on increased risk of pregnancy with concomitant implant and efavirenz use, and expanding contraceptive method mix for all women. As global access to ART expands, greater research is needed to explore implant effectiveness when used concomitantly with newer ART regimens. Data on how HIV-positive women and their partners choose contraceptives, as well as information from providers on how they present and counsel patients on contraceptive options are needed to help guide policy and service delivery. Lastly, greater collaboration between HIV and reproductive health experts at all levels are needed to develop successful strategies to ensure the best HIV and reproductive health outcomes for women living with HIV.

## Introduction

Up to 62% of pregnancies among women living with HIV in sub-Saharan Africa are unintended, contributing to HIV-related maternal morbidity and vertical HIV transmission [1–8]. Modern contraception, including hormonal and non-hormonal contraceptives, can help prevent unintended pregnancies. Paralleling a global shift, a marked increase in modern contraceptive prevalence has occurred in sub-Saharan African in recent decades, with average prevalence ranging from 8% in the 1980s to 22% in 2010 [9,10]. Long-acting reversible contraception (LARC), including progestin-containing subdermal implants and intrauterine devices (IUDs), are preferred by the World Health Organization (WHO) [11], and implants are the most effective LARC with increasing use in sub-Saharan Africa, including among HIV-positive women [9,10,12–14].

Though the expected contraceptive failure rate with implants is below 1% [15–19], drug–drug interactions between implants and antiretroviral therapy (ART), particularly efavirenz-based ART, may compromise implant effectiveness. Implant progestins, etonogestrel and levonorgestre, are metabolized by a hepatic cytochrome P450 enzyme, and efavirenz, a non-nucleoside reverse transcriptase inhibitor (NNRTI), is a potent inducer of this enzyme. Therefore, reduced implant effectiveness is thought to be related to lower systemic progestin concentrations [20,21]. This issue is particularly important because efavirenz-based ART remains the recommended first-line regimen by the WHO [22].

Below we present four inter-related perspectives on this issue. The first perspective examines a case study of how, based on limited data, authorities in South Africa recommended against the use of implants for women concomitantly using efavirenz, and how this policy led to unintended negative consequences. The second and third perspectives summarize the current clinical and pharmacokinetic (PK) data regarding concomitant implant and efavirenz-based ART use. The final perspective discusses the global implications of these findings for family planning and HIV programmes. We conclude with specific research recommendations that will aid providers and policy makers in helping HIV-positive women actualize their reproductive intentions with appropriate information, while maintaining options for highly effective family planning alongside ART.

## Discussion

### A case study: impact of country-specific guidance recommending against the use of implants for women prescribed efavirenz-based ART in South Africa

The implant was introduced into South Africa in early 2014, as part of a major revision of South Africa’s contraceptive guidelines, including an expanded contraceptive method mix that emphasizes LARC. By the start of 2015, nearly 900,000 implants had been inserted [23].

The 2012 National Contraceptive Guidelines advised that, despite potential drug–drug interactions between implants and certain ART, implants could be used by all ART users, particularly with concurrent condom use for enhanced pregnancy protection. In October 2014, emerging PK [24] and clinical pregnancy [25] data, coupled with reported implant failure among efavirenz users in South Africa, led the national Essential Medicines List Committee to change prescribing guidelines to: “women who are taking enzyme inducing drugs [such as efavirenz]…should not use progestin subdermal implants” [26]. This guidance was expanded upon in December 2014 allowing for continued concomitant implant and efavirenz use among women with an implant already inserted, “if the woman has been properly counseled” [27].

This guidance was intended to urgently inform providers about the possibility of decreased implant effectiveness when combined with efavirenz. However, an ongoing qualitative study in Western Cape Province, conducted by one of the co-authors (C.M.), revealed discontinuation of implant provision to most HIV-positive women, regardless of ART regimen, implant removals among women on efavirenz-based ART, and lack of support for efavirenz users who continued implant use. This study has documented several provider concerns, including risk of implant insertion in all HIV-positive women in case of future ART need, lack of providers’ understanding of specific drug–drug interactions, provider uncertainty in communicating this information to their patients, and a sense of clinical and legal vulnerability among the providers for potential implant failures. These results suggest that implants could be inadvertently removed as a contraceptive option for HIV-positive women in South Africa. In light of such unintended consequences, in the next two sections, we will review the current clinical and PK data, respectively, to more comprehensively understand the risk of implant failures with concomitant efavirenz-based ART use.

### Clinical data on contraceptive failures with concomitant implant and efavirenz use

Initially, case reports raised concerns of contraceptive failure when implants were combined with efavirenz-based ART [28–31]. The first clinical study of etonogestrel implants in Brazil found no pregnancies in the 20 women on efavirenz-based ART after three years of follow-up [32]. The second clinical study from Swaziland, a retrospective chart review of 332 ART users on levonorgestrel implants found that 15 (12.4%) of 121 women on efavirenz-based ART became pregnant in comparison to no pregnancies in the non-efavirenz-based ART groups [25]. The third clinical study combined data from three longitudinal studies and found that among implant users, the rate of incident pregnancy was 6.0 vs. 1.4 pregnancies/100 women-years in women using efavirenz-based ART vs. no ART, respectively [33].

Finally, a 3-year retrospective cohort study from Kenya examined pregnancy rates among nearly 25,000 HIV-positive women reporting use of various combinations of contraceptive methods and ART regimens [34]. Among implant users, they found the adjusted pregnancy rate was 3.3 per 100 women-years with efavirenz-based ART, compared with 1.1 with nevirapine-based ART and 1.3 with no ART. In other words, among women using implants, those using efavirenz-based ART had three-fold higher pregnancy rates than women using nevirapine-based ART (95% CI 1.3–4.6). However, this study also showed that among efavirenz-based ART users, women reporting use of oral and injectable contraceptives were up to three times more likely to become pregnant than those reporting use of implants (adjusted pregnancy rate ratio of 2.8 (0.97–4.7) and 1.6 (0.83–2.5), respectively). Hence, the Kenya study suggests that despite drug–drug interactions, implants remain more or as effective than oral and injectable contraceptives among HIV-positive women using efavirenz.

The cohort studies each have unique limitations, such as self-reported contraceptive use or a small sample size. The study from Brazil was limited by a small sample size, follow-up every 6 months potentially leading to missed early pregnancy loss, low adherence to ART, and lack of clarity of how many participants reached the 3-year end point. Several limitations affected the Kenyan study, which offers the most comprehensive data on this topic. First, since contraceptive use was self-reported, social desirability for using contraception and difficulties discussing fertility intentions with providers may have led women to more often report contraceptive use, particularly methods such as injectables that could not be verified by providers. Second, pregnancy was diagnosed clinically, thus potentially leading to underreported early gestation pregnancy loss. Last, though the authors adjusted for concurrent condom use, other potential confounders, such as adherence to both contraceptive method and ART and sexual activity could not be adjusted for in this current analysis.

Future prospective studies that include frequent ascertainment and independent verification of contraceptive use, ART regimens, and pregnancy can overcome the limitations of the existing cohort studies. However, such prospective studies will require long periods of follow-up and be costly. Therefore, a role remains for efficiently combining findings from PK studies with secondary analyses of routinely collected clinical data. As such, in the next section, we elaborate on the existing PK evidence regarding concomitant implant and efavirenz use.

### PK of implant progestins in combination with efavirenz-based ART

Pharmacologic evidence to support the biologic basis for reduced effectiveness of implants in women on efavirenz has emerged from four recent PK studies. Three prospective studies inserted implants at study entry and compared plasma progestin concentrations in women receiving efavirenz-based ART to a similar group of HIV-positive women not receiving ART (control group). The first study assessed etonogestrel PK for 24 weeks after implant insertion (*n* = 14 per group) [24,35]. At week 24, etonogestrel concentrations were 70% lower in the efavirenz group, compared to the control group (69 vs. 230 pg/mL; *p* < 0.001). Luteal activity, a surrogate marker of the ability to become pregnant, occurred in 2.8–5% of participants receiving efavirenz compared to no subject in the control group (*p* < 0.05). A similar study was conducted in Uganda that described levonorgestrel PK over 48 weeks (*n* = 20 per group) [35,36]. At week 48, levonorgestrel concentrations were 57% lower in the efavirenz group, compared to the control group (247 vs. 580 pg/mL; *p* < 0.001). Between study weeks 36 and 48, three women (15%) in the efavirenz group became pregnant. No pregnancy occurred in the control group. Another study in Uganda found 82% lower etonogestrel concentrations after 24 weeks of the implant plus efavirenz-based ART in 19 women compared to 20 women in the control group (66 vs. 362 pg/mL, *p* < 0.001); all women receiving efavirenz in this study also had a copper IUD in place, so no pregnancies were observed [37]. In addition to these prospective cohorts, one cross-sectional study evaluated etonogestrel concentrations 6–7 weeks after implant insertion in women receiving efavirenz- (*n* = 9) or protease inhibitor-based ART (*n* = 45) [38]. Women receiving efavirenz had >90% lower etonogestrel concentrations (41.5 pg/mL) compared to those receiving protease inhibitors. In addition, the authors report one pregnancy (11%) 16 months after implant insertion in a woman receiving efavirenz.

Overall, these PK results for efavirenz plus implant progestins are consistent with efavirenz-progestin interactions observed with oral and transdermal routes of contraceptive administration [20]. Notably, nevirapine, another NNRTI, did not adversely influence implant progestin exposure [20,37]. These results are consistent with reports of nevirapine plus oral progestins [20], suggesting that nevirapine has less potent cytochrome P450 enzyme induction than efavirenz. Finally, the effect of lopinavir/ritonavir and atazanavir/ritonavir on etonogestrel were evaluated in two of the studies [24,38] and higher progestin exposure was observed without excess adverse events, suggesting that contraceptive implants are not adversely effected by protease inhibitors.

While assessing drug concentrations is a standard approach to measure the expected influence of drug–drug interactions on clinical outcomes [39], the implant PK studies to date were not designed to concurrently evaluate contraceptive effectiveness. In addition, these were nonrandomized, observational studies, due to the clinical necessity of ART initiation and long follow-up required to characterize implant progestin PK. Despite these limitations, three of the four studies directly observed pregnancies, a clinically significant outcome. Further, the proposed implant progestin PK threshold for contraceptive effectiveness is based on few cases of observed pregnancies. For example, 180 pg/mL was previously suggested as the desired levonorgestrel threshold based on five pregnancies in women receiving the implant for longer than its intended duration of use [40]. In contrast, two of the three pregnancies observed in the Ugandan study occurred above this threshold (297 and 303 pg/mL) [35]. This may be related to the anticipated variability when observing few cases or due to different laboratory methodologies. Nonetheless, the current PK data are consistent with each other and the clinical data. Future PK studies also investigating measures of contraceptive effectiveness, such as cervicovaginal mucus or ovulation, may help better establish thresholds for contraceptive effectiveness; however, such studies will be time and resource intensive, again highlighting the importance of combining clinical and PK data.

### Implications for global family planning and HIV programmes and policies

In light of the clinical and PK data, we now examine the implications of such data for family planning and HIV programmes and policies globally, given the growing population of women using concomitant contraceptive implants and ART. First, integrating family planning services into existing HIV platforms provides an important opportunity to reach providers and women with information about how these medications may interact. This provides women the opportunity to obtain counselling with the same provider for both her family planning and HIV needs, streamlining holistic care for the women.

Second, an implant being a more effective method than other hormonal methods when used concomitantly with efavirenz does not negate the fact that implants are failing for some women. HIV-positive women considering implants should be informed about the possibility of the implant’s decreased effectiveness with concomitant efavirenz use. Counselling on the potential risks of failure is essential to ensure that women can consider other effective methods, and if they choose implants, recognize signs of early pregnancy. Nonetheless, women should retain the right to make an informed decision and still choose implants after weighing the risks and benefits of available methods.

Last, the challenge of concomitant implant and efavirenz use highlights the necessity of a robust contraceptive method mix, which includes more choices for LARC. For example, non-hormonal IUDs could be a potential alternative to implants. However, persistent challenges to optimizing method mix include method cost, provider preferences, lack of trained staff, weak logistics and supply management, lack of demand for certain methods, and misinformation. Donor and country partners are supporting efforts to address these challenges and provide a range of commodities to ensure HIV-positive women have a real choice when weighing contraceptive risks and benefits relevant for their lives.

## Conclusions

As global access to ART expands, there are critical gaps in evidence to support the combination of implants with newer ART regimens. Existing clinical data indicate reduced contraceptive effectiveness when implants and efavirenz are used concomitantly, though the implants still remain more effective at preventing pregnancy than most alternative hormonal contraceptive methods. PK studies that find significantly reduced levonorgestrel and etonogestrel concentrations when used with efavirenz demonstrate the biologic mechanism of reduced implant efficacy. While PK and clinical data may support the combination of nevirapine [35,37], atazanavir/ritonavir- [38,41], or lopinavir/ritonavir-based ART [24,38] with implants, these regimens are not recommended as first-line regimens for HIV treatment due to issues of side effects or resistance profiles [42–45]. Research on the combination of implants with newer ART regimens, such as dolutegravir-based ART, could provide critical information prior to or during early rollout of new treatment guidelines.

Efavirenz-based ART will continue to be a first-line regimen in most resource-limited settings for the foreseeable future, particularly in combination with anti-tuberculosis treatments. Therefore, data to support alternative strategies for optimizing the effectiveness of contraceptive implants in combination with efavirenz-based ART are urgently needed. Such strategies could include increasing the implant dose to maintain blood concentrations above a threshold of efficacy or decreasing the length of implant use if failures are related to increasing duration of implant use. Data regarding the influence of reducing the efavirenz dose from 600 mg to 400 mg/day on contraceptive hormone drug–drug interactions are also urgently needed.

Finally, a single, cross-over, PK study with short-term oral contraceptives in healthy subjects are currently required by regulatory authorities, such as the United States Food and Drug Administration, to characterize a new drug’s impact on hormonal contraceptive exposure. This scenario with implants and efavirenz could have been identified and addressed earlier if pharmaceutical companies were required to conduct more comprehensive PK testing with several hormonal contraceptive methods, given the diversity of hormonal contraceptive hormones and routes of administration. Finally, simply providing impractical statements that barrier methods should always be used in conjunction with hormonal contraception does not provide appropriate options for women living with HIV [46].

Ultimately, greater collaboration between HIV and reproductive health experts at all levels are needed to develop successful strategies to ensure the best HIV and reproductive health outcomes for women living with HIV. South Africa’s guidance implementation provides important lessons on why collaboration is essential. To facilitate this, information on values and preferences with respect to how HIV-positive women and their partners choose contraceptives, as well information from providers on how they present and counsel on contraceptive options are needed to help guide policy and service implementation. A focus on developing appropriate information and counselling tools to support providers and HIV-positive women is needed to avoid blanket guidance against concomitant implant and efavirenz use. Finally, using a rights-based approach to this issue can help ensure all women living with HIV are able to choose a contraceptive method that is best suited for their lives from among a range of options.
